# Woodhouse-Sakati Syndrome: The New Genetic Variant of DCAF17 In 2 Adult Sisters

**DOI:** 10.1210/jcemcr/luae130

**Published:** 2024-07-24

**Authors:** Maria Amosova, Irina Poluboyarinova, Valentin Fadeev, Aliy Asanov

**Affiliations:** Endocrinology Department, Sechenov University, 119991 Moscow, Russia; Endocrinology Department, Sechenov University, 119991 Moscow, Russia; Endocrinology Department, Sechenov University, 119991 Moscow, Russia; Department of Medical Genetics, Sechenov University, 119991 Moscow, Russia

**Keywords:** alopecia, diabetes mellitus, Woodhouse-Sakati syndrome, novel mutation

## Abstract

Woodhouse-Sakati syndrome (WSS) is a rare autosomal recessive disorder associated with progressive extrapyramidal signs, mental retardation, alopecia, and a variety of endocrine deficiencies, including diabetes mellitus, hypogonadism, and hypothyroidism. To date, approximately 98 genetically confirmed WSS families have been reported worldwide. This report focuses on a new genetic variant detected in 2 WSS-affected sisters with distinctive phenotypical features. The case under review is of special interest due to the multiple manifestations of WSS. This is the first family case of WSS identified in the Russian Federation. Although there is no specific treatment for WSS, genetic testing makes it possible to diagnose WSS, make a prognosis, and provide comprehensive patient-oriented treatment.

## Introduction

Woodhouse-Sakati syndrome (WSS) is a rare autosomal recessive disorder. N. Woodhouse and N. Sakati observed the condition in several Arab families and first described it in 1983 (1). In 2007-2008, a common mutation in the *DCAF17* (*C2orf37*) gene was identified in these families, which was followed by the appearance of several molecularly verified cases of various origins (Arabian, Turkish, Italian, Indian, Serbian) (2). Since 2008, approximately 187 patients (98 families) have been described (3, 4). The main features of WSS are listed in Table 1 (5). The exact mechanism of WSS pathogenesis remains unclear; however, the disease is thought to be a possible result of nucleolar malfunction affecting the regulation of the cell cycle or cell aging (2).

**Table 1.** Presence of the main WSS signs in the sisters and their incidence among all WSS cases (5)

Signs	Presence in the sisters		Incidence (%) in patients with WSS described previously
	Sister A. 18 years old	Sister M. 29 years old	
Alopecia totalis	+	+	100
Diabetes mellitus	+	+	66
Neurosensory hypoacusis	+	+	62
Hypogonadism	+	+	100
Mental retardation	+ Mild cognitive impairment	+ Corresponds to the level of development according to the interview	53
Dental decay	+	+	10
Low level of IGF-1	+	+	30
Hypothyroidism	—	+	30
Lesions of white matter	+ Headache; dyslexia, dysgraphia	+ Headache	Reported in some cases
Polyneuropathy	+	+	Reported in some cases
Dysmorphic facial features	+ Asymmetry of the odontoid process relative to the lateral masses; limited motility of the lower jaw	+ A small lower jaw, an asymmetric nasolabial triangle	Reported in some cases
ECG (inverted/flattened T waves)	+	+	Reported in some cases
keratoconus, anodontia	—	—	reported in some cases

This work describes the clinical case of the 2 sisters with WSS related to a c1422 + 3G > T mutation in the *DCAF17* gene. This genetic variant has not been previously described. This is also the first case of WSS identified in the Russian Federation. Both sisters had diabetes mellitus, hypergonadotropic hypogonadism, alopecia, neurosensory hypoacusis, and moderate mental retardation. This combination of endocrine, dermatological, and neurological symptoms is typical of WSS. The diagnosis was corroborated through the utilization of molecular genetic testing, which identified a variant in the DCAF17 gene.

In fact, WSS is a rare disease, and reporting new cases makes it possible to obtain more data and as a result describe the syndrome in more detail and diagnose it earlier. Diagnosis verification makes it possible to make justified decisions on appropriate treatment methods and further medical and genetic counseling of the concerned families.

## Case Presentation

### Case 1

**Figure 1.**
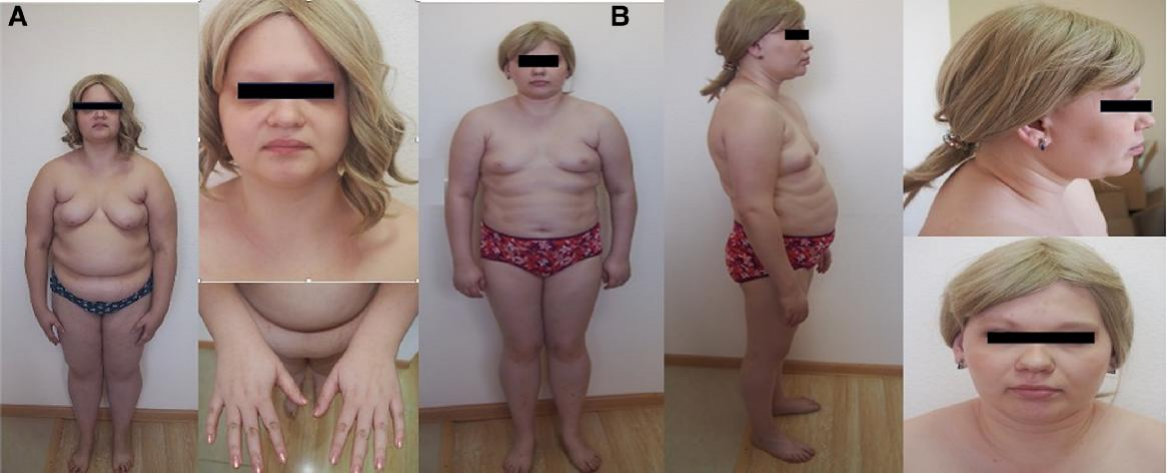
(A, Case 1 and B, Case 2) Photographs of the sisters with WSS. Note the dysmorphic face, alopecia (sisters wear wigs), bilateral thumb hypoplasia, and a dowager's hump.

A 18-year-old woman presented with a 4- to 5-year history of amenorrhea, diffuse alopecia, obesity, and diabetes (controlled with metformin 1500 mg/daily). Oligomenorrhoea was reported since menarche at age 13 years, followed by secondary amenorrhea. She also reported hearing loss. In her teens, she was periodically homeschooled. Currently, she is attending a college for students with special needs. Clinical examination showed an increase body mass index (BMI) of 30.38 kg/m^2^ (height 155 cm, body weight 73 kg), additional adipose tissue accumulation over VI cervical vertebrae and marked WSS-related features (Table 1), also pale-pink skin striations in the abdominal area. Secondary sexual characteristics were underdeveloped with Tanner stage 2 breasts and pubic hairs. We also noted camptodactyly which involves the fixed flexion deformity of the interphalangeal joints of the fifth finger (shown in Fig. 1A). Given the presence of specific complex clinical signs and features consistent with WSS, the patient was suspected of having WSS.

### Case 2

A 29-year-old woman was referred with the signs of WSS (Table 1). Her medical history included alopecia and amenorrhea since the age of 12; diabetes diagnosed at 16 (requiring basal-bolus insulin therapy—glargine, glulisine, with glycemic control goals not achieved); and hearing impairment started at age 17. During childhood, she met all developmental milestones on time, exceling both intellectually and physically. After completing secondary school, she earned a registered nurse degree and worked in an outpatient clinic, showing superior cognitive abilities compared to her sister. Her height was 152 cm, and her body weight was 63 kg. Other findings included excessive subcutaneous body fat with abdominal distribution and camptodactyly of the fifth finger on each hand. The development of her external genitalia was in conformity with her age; secondary sexual characteristics were underdeveloped, with Tanner stage 2 breasts and pubic hairs (shown in Fig. 1B).

## Diagnostic Assessment

### Case 1

Examination in our clinic (Table 2) revealed that levels of prolactin, thyrotropin (thyroid-stimulating hormone [TSH]), free thyroxine (T4), and adrenocorticotropic hormone (ACTH) levels were within the reference range, with a mild decrease in her insulin-like growth factor 1 (IGF-1) level. Hypergonadotropic hypogonadism was diagnosed. The insulin hypoglycemia test excluded both growth hormone deficiency and adrenal insufficiency. Biochemical investigations also showed increased glycated hemoglobin at 9.9% (85 mmol/mol) with intact pancreatic beta cell function. The assessment of potential late complications of diabetes mellitus for the first time revealed the presence of microalbuminuria at 33 mg/day (normal range, 0-30) alongside a reported glomerular filtration rate of 73.21 mL/min/1.73 m^2^ (CKD-EPI) what requires further confirmation. No signs of diabetic retinopathy or peripheral neuropathy were found. Blood pressure remained in the range of 110-120/70-80 mm Hg, and the heart rate was 60 to 75 per minute. Electrocardiography (ECG) reflected flattened T waves. The audiogram results revealed neurosensory hypoacusis. Pelvic ultrasound revealed uterine hypoplasia with normal ovaries. Brain magnetic resonance imaging (MRI) showed a normal size and shape of the pituitary gland and reveals small foci of gliosis in the frontal, parietal, and occipital lobes on both sides.

**Table 2.** The sisters’ laboratory examination results during hospitalization in our clinic

Laboratory analysis	Case 1	Case 2	Reference range
HbA1c	9.9% (85 mmol/mol)	9.8% (84 mmol/mol)	6.5%-7.0% (47.5-53 mmol/mol)
Basal C-peptide	758 pmol/L (2.3 ng/mL)	533 pmol/L (1.6 ng/mL)	298-2 350 pmol/L (1.1-4.4 ng/mL)
Stimulated C-peptide at 2 hours after the glucose load test	834 pmol/L (2.5 ng/mL)	755 pmol/L (2.3 ng/mL)	298-2350 pmol/L (1.1-4.4 ng/mL)
LH	25.2 mIU/mL (25.2 IU/L)	17.9 mIU/mL (17.9 IU/L)	1.5-12.5 mIU/mL (1.5-12.5 IU/L)
FSH	50.2 mIU/mL (50.2 IU/L)	41.1 mIU/mL (41.1 IU/L)	1.4-10.1 mIU/mL (1.4-10.1 IU/L)
Estradiol	55 pmol/L (15 pg/mL)	48 pmol/L (13 pg/mL)	72-529 pmol/L (20-400 pg/mL)
TSH	3.2 mIU/L (3.2 IU/L)	5.6 mIU/L (5.6 IU/L)	0.4-4.0 mIU/L (0,4-4,0 IU/L)
Free T4	1.01 ng/dL (13.1 pmol/L)	0.65 ng/dL (8.4 pmol/L)	0.7-1.8 ng/dL (11.5-23.2 pmol/L)
TPO-Ab	neg	neg	neg
IGF-1	111 ng/mL (14.5 nmol/L)	114 ng/mL (15 nmol/L)	176-429 ng/mL (20-50 nmol/L)
ACTH	36.4 pg/mL (8 pmol/L)	64.1 pg/mL 14.1 pmol/L	10-60 pg/mL (0-10.2 pmol/L)
Cortisol (basal)	26.4 mcg/dL (728 nmol/L)	22 mcg/dL (607 nmol/L)	5-25 mcg/dL (119-618 nmol/L)
Cortisol (hypoglycemia)	31.3 mcg/dL (864 nmol/L)	24.8 mcg/dL (684 nmol/L)	> 20 mcg/dL. (>550 nmol/L)
GH (basal)	0.8 mIU/L (0.26 ng/mL)	1.2 mIU/L (0.4 ng/mL)	0,16-13mIU/L (0.4-10 ng/mL)
GH (hypoglycemia)	10.8 mIU/L (3.6 ng/mL)	10.1 mIU/L (3.3 ng/mL)	>9 mIU/L (>3 ng/mL)

Values in parenthesis are in International System of Units (SI).

Abbreviations: ACTH, adrenocorticotropic hormone; FSH, follicle-stimulating hormone; GH, growth hormone; HbA1c, glycated hemoglobin; IGF-1, insulin-like growth factor 1; LH, luteinizing hormone; T4, thyroxine; TPO-Ab, thyroid peroxidase antibodies; TSH, thyroid-stimulating hormone.

### Case 2

Endocrine workup in our clinic (Table 2) excluded growth hormone deficiency and adrenal insufficiency but confirmed hypergonadotropic hypogonadism. A slightly elevated TSH level associated with decreased free T4 and absence of thyroid peroxidase antibodies (TPO-Ab) suggested the development of secondary hypothyroidism. Sufficient secretion of basal and stimulated C-peptide levels was evident. Diabetic complications included proteinuria with a normal glomerular filtration rate and proliferative diabetic retinopathy OU, and diabetic neuropathy with decreased vibration and temperature sensitivity. An ECG showed inverted T waves. Pelvic ultrasound revealed a hypoplastic uterus and not visualized ovaries, and breast ultrasonography found no differentiated glandular tissue. Neurosensory hypoacusis was confirmed through an audiogram. MRI revealed pituitary flattening with distinguishable lobes. MRI signs of vascular lesions in the white matter of the brain—cerebral microangiopathy.

### Genetic study

**Figure 2.**
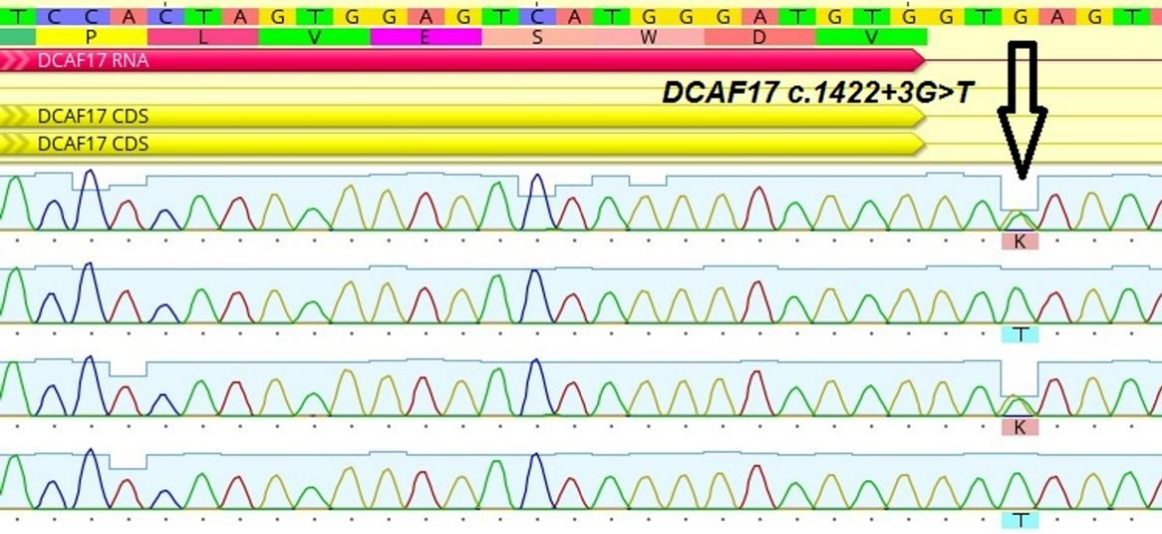
Sanger sequencing chromatogram from probands and his parents. Father, Proband 1, Mother, Proband 2.

Taking into account the pattern of specific and typical phenotyping WSS signs in the family, which did not require differential diagnosis with other possible genetic syndromes, a molecular genetic study was performed. Blood samples were collected from both patients and parents, and genomic DNA was extracted from blood cells using a standard procedure. All coding exon sequences and exon-intron sequences of the *DCAF17* gene boundaries were obtained by PCR amplification and sequencing of PCR products on a genetic analyzer. The method for direct automated sequencing was used to examine exons 04 and 13 of the *DCAF17* gene as well as adjacent intronic regions. In both sisters, a genetic variant NM_025000.3: c.1422 + 3G > T in intron 13 of the *DCAF17* gene was found in the homozygous state (shown in Fig. 2). Computer-assisted analysis (Alamut visual, version 2.10) predicted that the gene variant might result in intron 13 splicing impairment and most likely induce skipping of exon 13, also confirming pathogenicity. In both parents, a genetic variant c.1422 + 3G > T in intron 13 of the *DCAF17* gene was found in the heterozygous state (shown in Fig. 2).

According to the Human Gene Mutation Database (HGMD Professional), 19 pathogenic variants in the *DCAF17* gene have been described in patients with WSS and hypergonadotropic hypogonadism. The HGMD Professional database does not mention any extended deletions or duplications in this gene. The nucleotide variant identified in our patients has not been previously described. However, the c.1422 + 5G > T variant has been reported in patients with WSS by authors from India (2, 6). The c.1422 + 3G > T variant is predicted to cause splicing disorders according to computer analysis (Alamut Visual Plus, version 1.2.1). Additionally, it should be considered as likely pathogenic variant based on family segregation analysis according to American College of Medical Genetics and Genomics criteria (ACMG evidence codes used: PVS1, PM2_supporting, PP3_supporting, PP1 supporting).

## Treatment

### Case 1

We initiated combined hormone replacement (estrogen-progestogen) therapy for hypergonadotropic hypogonadism. Due to decompensated diabetes mellitus and intact pancreatic beta cell function, glibenclamide was prescribed as an alternative to metformin.

### Case 2

Replacement hormonal therapy for hypergonadotropic hypogonadism was prescribed. Since we observed sufficient secretion of basal and stimulated C-peptide levels, basal insulin therapy was substituted with the sodium glucose co-transporter 2 (SGLT2) inhibitors empagliflozin and metformin at daily doses of 25 mg and 2000 mg, respectively.

## Outcome and Follow-Up

Treatment of diabetes in both effectively achieved the target glucose levels of 5.0 to 7.0 mmol/L (90-126 mg/dL) fasting and up to 8 mmol/L (144 mg/dL) during the day. No hypoglycemic episodes were noted. After a short period of use, they decided to discontinue estrogen-progestogen replacement therapy.

### Case 1

Upon subsequent follow-up, microalbuminuria was confirmed with chronic kidney disease stage 2.

### Case 2

Follow-up assessment confirmed a reduction of free T4 and a normal TSH level. The presentation was characterized as secondary hypothyroidism within the context of WSS, prompting the initiation of L-thyroxine at a dosage of 75 mcg.

## Discussion

The spectrum of lesions in WSS predominantly involves the neuroendocrine system. Quite typically, as noted in the present case, hypogonadism is the first manifestation of endocrine deficiency. Hypogonadism is distinctive by mixed origin. Some literature describes hypergonadotropic, hypogonadotropic, and combined hyper/hypogonadotropic hypogonadism. However, women tend to develop hypergonadotropic hypogonadism. This endocrine picture might be the result of the absence of functional ovarian tissue. Due to hypergonadotropic hypogonadism, we prescribed estrogen-progestogen replacement therapy for both our patients; however, they subsequently discontinued this treatment.

Isolated low IGF-1 levels have been described in all patients with WSS. In our case, a low IGF-1 level was exclusively observed in both sisters. The absence of clinical signs of low IGF-1 as well as the results of the insulin hypoglycemia test, demonstrated the absence of somatotropic insufficiency in this patient, which suggests another mechanism of the lesion that does not affect the somatotropic axis. The exact pathophysiology of decreased IGF-1 has not yet been determined.

To date, no abnormalities of the corticotropic axis have been described. We also did not find any impairments of this axis in our patients.

The alterations in TSH production and thyroid hormone levels found in the elder sister are of particular interest. Thyroid ultrasound did not reveal any structural abnormalities of the gland. Mild elevation of TSH associated with low free T4 levels and the absence of antithyroid antibodies may indicate the development of secondary hypothyroidism. During the follow-up assessments, a persistent decrease in free T4 levels led to the initiation of levothyroxine replacement therapy.

Diabetes mellitus is reported in 66% of all patients with WSS. Almost all patients develop diabetes by the age of 20. Considering anamnestic and clinical data, we can assume that diabetes in such cases is not autoimmune in nature. Most likely, the disease is associated with progressive deterioration in pancreatic β-cell function. Moreover, no cases of diabetic ketoacidosis have been registered to date. It is not known why pancreatic β-cells are damaged. Taking into account the nonautoimmune nature of diabetes, we selected an individual regimen of hypoglycemic therapy for each patient. Sufficient C-peptide levels, which reflect preserved insulin secretion, made it possible to prescribe sulfonylurea agents with a good therapeutic effect in the younger sister. The elder sister was switched from previously prescribed basal-bolus insulin therapy to an SGLT2 inhibitor in combination with metformin and basal insulin, thus significantly decreasing the variability of glycemia. Finally, we noted the same ECG changes in our patients as described in other cases of WSS. However, no structural impairments of the heart have been detected.

Although there exists no specific treatment for WSS, genetic testing confirms the diagnosis, makes possible a more accurate prognosis and provides a comprehensive personalized approach to treatment.

## Learning Points

- Our case highlights that WSS is a rare autosomal recessive disorder with a variety of endocrine, dermatological, and neurological symptoms.
- In patients with diabetes, obesity, alopecia, hypergonadotropic hypogonadism, and neurosensory hypoacusis associated with neurological symptoms, WSS syndrome should be considered with appropriate investigation and DCAF17 genetic testing for the patient and family members.
- If WSS syndrome is suspected, a full examination is necessary to exclude all possible components of the syndrome (the list is presented in this article).
- Also of particular interest are the features of the treatment of diabetes mellitus in patients with WSS as well as the possible presence of secondary hypothyroidism not previously described.
- Genetic testing makes possible a more accurate prognosis and provides a comprehensive personalized approach to treatment.

## Data Availability

Original data generated and analyzed for this case report are included in this published article.

## Abbreviations

BMI: body mass index
ECG: electrocardiography
IGF-1: insulin-like growth factor 1
MRI: magnetic resonance imaging
SGLT2: sodium glucose co-transporter 2
T4: thyroxine
TSH: thyrotropin (thyroid-stimulating hormone)
WSS: Woodhouse-Sakati syndrome
